# Replacing Fish Meal with Spirulina (*Arthrospira platensis*): Nutrigenomic Modulation of Growth, Reproductive Performance, and Metabolism in Zebrafish

**DOI:** 10.3390/ani15172552

**Published:** 2025-08-30

**Authors:** William Franco Carneiro, Pamela Navarrete-Ramírez, Tassia Flávia Dias Castro, Estéfany Ribeiro Leão, Carlos Cristian Martínez-Chávez, Carlos Antonio Martínez-Palacios, Luis David Solis Murgas

**Affiliations:** 1 Departamento de Medicina Veterinária, Universidade Federal de Lavras, Lavras 37200-000, MG, Brazil; willfc14@gmail.com; 2 SECIHTI—Instituto de Investigaciones Agropecuarias y Forestales, Universidad Michoacana de San Nicolas de Hidalgo, Morelia 58330, Mexico; pnavarretera@conahcyt.mx; 3 Instituto de Ciências Biomédicas da Universidade de São Paulo (ICB-USP), São Paulo 05508-000, SP, Brazil; tassia_fd@hotmail.com; 4 Escola de Ciências Agrárias de Lavras, Departamento de Agricultura, Universidade Federal de Lavras, Lavras 37200-000, MG, Brazil; estefany.leao1@estudante.ufla.br; 5 Instituto de Investigaciones Agropecuarias y Forestales, Universidad Michoacana de San Nicolas de Hidalgo, Morelia 58330, Mexico; cmartinez@umich.mx (C.C.M.-C.); cpalacios@umich.mx (C.A.M.-P.)

**Keywords:** microalgae, aquaculture nutrition, RNA-Seq, transcriptomics

## Abstract

Protein-rich microalgae have been increasingly recognized as viable alternatives to fish meal (FM) in aquaculture diets. In this study, we evaluated the effects of partial or total replacement of FM with the microalga *Arthrospira platensis* (Spirulina, SM) on the growth performance, reproductive parameters, and transcriptomic profile of zebrafish. Six isoproteic, isoenergetic experimental diets were formulated with increasing levels of SM (0, 10, 20, 30, 40, and 50 g kg^−1^ feed) replacing FM. Fish were randomly assigned to six groups (five replicates each) and fed for 60 days. The diet containing 50 g kg^−1^ SM resulted in the highest final body weight, weight gain, specific growth rate, and protein efficiency, as well as increased gonadosomatic index, eggs per female, fertilization rate, and hatching rate compared to the results for the control group (0 g kg^−1^ SM). RNA-Seq transcriptomic analysis identified 2299 differentially expressed genes in the SM50 group, mainly associated with muscle development and energy metabolism. These findings offer new insights into the underlying molecular mechanisms and underscore the potential of Spirulina as a sustainable alternative for cultured fish nutrition.

## Simple Summary

The increasing use of fish meal in the diets of captive-reared fish has raised environmental and economic concerns, since this practice is not sustainable in the long term. Therefore, it is important to find alternative ingredients that are both efficient and sustainable. In this study, we evaluated whether Spirulina, a protein-rich microalga, could replace fish meal in the diet of zebrafish, a species frequently used in scientific research. We found that completely replacing fish meal with Spirulina improved fish growth and reproductive capacity, resulting in larger individuals with higher egg production. Furthermore, we discovered that this substitution upregulated genes related to muscle growth and energy metabolism. These results demonstrate that Spirulina can be successfully used in the nutrition of farmed fish, providing benefits for the environment by reducing the use of scarce natural resources and enhancing the productive performance of the fish.

## 1. Introduction

Fish meal (FM) is widely used as a protein source in aquafeeds due to its nutritional profile, which includes essential amino acids, fatty acids, and minerals, as well as its low levels of antinutritional factors [1,2]. However, although global production remains relatively stable, it still does not fully meet the growing demand of the aquaculture industry, leading to the need for alternative protein sources in formulated diets [3,4]. The increasing pressure on marine resources, together with the fluctuating prices of fish meal and uncertainties in the supply chain, have intensified the urgency to identify sustainable and economically viable protein alternatives [4]. Therefore, new protein sources are required as FM substitutes to ensure sustainable aquaculture development. In this context, microalgae are considered potential alternatives to FM due to their high protein content and well-balanced amino acid profile [5]. In addition, microalgae offer several advantages over conventional protein sources, such as rapid growth rates, minimal land requirements, and the ability to be cultivated in various environments without competing with other food crops [6]. In addition, microalgae can be produced year-round under controlled conditions, providing a more predictable and sustainable supply chain [7].

One of the most promising alternatives is the microalga *Arthrospira platensis* (Spirulina), which belongs to the family Oscillatoriaceae and is widely found in both freshwater and saltwater environments [8]. Spirulina has a high protein content (55–70%), an adequate amino acid profile, and contains essential fatty acids, as well as displaying strong antioxidant activity and immunomodulatory properties, regulating important inflammatory cytokines such as IL-1β, IL-6, IL-10, and TNF-α and exerting modulatory effects on the antioxidant system [9,10,11,12,13]. In addition to its nutritional benefits, Spirulina contains unique bioactive compounds, including phycocyanin, chlorophyll, and carotenoids, which may provide additional health benefits [7].

Several studies have already demonstrated positive effects of the partial inclusion of Spirulina on growth and health in aquaculture species such as Nile tilapia (*Oreochromis niloticus*), common carp (*Cyprinus carpio*), and rainbow trout (*Oncorhynchus mykiss*) [14,15]. However, prior studies have concentrated predominantly on growth, yielding heterogeneous outcomes across species and formulations [7]. Reproductive parameters, particularly under high Spirulina inclusion or complete FM replacement, remain underexplored.

Although the general effects of Spirulina as an immunomodulator and on growth performance are known, the molecular mechanisms involved in fish response to FM replacement with Spirulina are not yet well understood. This molecular-level understanding is essential for optimizing diet formulations and predicting potential long-term effects of complete FM replacement. The advent of next-generation RNA sequencing (RNA-Seq) technology provides a valuable tool for elucidating the impact of specific nutritional components on gene expression and metabolic pathways in fish [16]. This knowledge can facilitate the development of more efficient and sustainable diets.

Additionally, the complexity of nutritional trials using traditionally farmed fish species creates economic and methodological challenges due to the high costs and long durations of the experiments. As a result, these constraints hinder the simultaneous evaluation of growth and reproductive parameters under controlled conditions. One strategy for overcoming these challenges is to use experimental models such as zebrafish (*Danio rerio*), which enables faster and less expensive studies in experimental aquaculture [16,17,18]. Zebrafish offer several advantages, including short generation times, well-characterized genetics, established reproductive protocols, and their suitability for high-throughput molecular analyses [19].

Therefore, this study aimed to evaluate the effects of replacing FM with Spirulina on growth, reproductive performance, and differential gene expression in zebrafish. We hypothesized that Spirulina substitution would modulate growth and reproduction via specific alterations in molecular pathways associated with nutritional and immune responses.

## 2. Materials and Methods

### 2.1. Ethical Statement

All experimental procedures used in this study were conducted in strict accordance with the institutional guidelines of the Animal Experimentation Ethics Committee of the Federal University of Lavras (UFLA), Lavras, MG, Brazil, under protocol no. 108/18, and complied with the guidelines of the National Council for the Control of Animal Experimentation (CONCEA) for the care and use of laboratory animals.

### 2.2. Origin and Maintenance of Zebrafish

Wild-type juvenile zebrafish, 30 days post-fertilization (dpf), were obtained from a local supplier and maintained at the Central Animal House of UFLA, where the experimental phases were conducted. From first feeding until the beginning of the trial, the fish were reared in a recirculating aquaculture system (RAS), as described by Carneiro et al. [20]. After two weeks of acclimation, juveniles with an average body weight of 160.14 ± 1.09 mg were transferred to the experimental system, consisting of thirty 3 L polycarbonate tanks (11.5 cm × 34.5 cm × 15.5 cm), arranged in a rack designed for the species (Rack Hidrus, model ZEB-40, Alesco, Monte Mor, Sao Paulo, Brazil). Fish were randomly assigned to experimental groups using a table of random numbers. The tanks were connected to an RAS with automated control of temperature, pH, and conductivity. Water from the tanks flowed by gravity to a sump-type filtration tank, equipped with three polypropylene bag filters for filtering particles of 100 µm and three felt filters for retaining particles of 50 µm. The water then passed through a biological filter containing BioBalls and ceramic rings and was pumped into a chamber with ultraviolet light before returning it was returned to the tanks. Throughout the growth trial, the fish were kept under artificial light with a 14 h light/10 h dark cycle. The average temperature was maintained at 27.8 ± 0.6 °C, the pH at 7.4 ± 0.3, and nitrogenous compounds remained at residual levels.

### 2.3. Experimental Diets

Six isoproteic (320 g kg^−1^ crude protein) and isoenergetic (17 MJ kg^−1^ gross energy) diets were formulated to contain different levels of FM replacement by *Arthrospira platensis* (SM) was used for diet supplementation (Commercial Spirulina Powder, Bernaqua, 72% protein). Up to 100% of FM was replaced with SM, with diets containing 0, 1, 2, 3, 4, and 5% SM. The crude protein requirement values were the same as those proposed by O’Brine et al. [21]. All diets were extruded in 4–6 mm extruders. They were then dried in a forced-air oven (55 °C/12 h), broken into appropriate sizes for the animals, and stored at −20 °C until use. The proximate composition of the diets and SM (Table 1) was analyzed according to AOAC methodology [22] for crude protein (Method 984.13), ether extract (Method 920.39), moisture (Method 930.15), and ash (Method 942.05), and gross energy was determined using a bomb calorimeter (Model-IKA C5000, IKA-Werke GmbH & Co. KG, Staufen, Germany).

Diets were randomly assigned to six groups, each group consisting of five replicates, with 15 fish per replicate. Fish were fed to apparent satiation three times daily (8:00 a.m., 11:00 a.m., and 3:00 p.m.) for a period of 60 days.

### 2.4. Growth and Reproductive Performance

At the end of the growth trial, all fish from each tank were removed, and after anesthesia via a benzocaine bath (250 mg L^−1^), they were individually weighed and measured. Final weight (FW), weight gain (WG), specific growth rate (SGR), feed efficiency (FE), daily feed intake (DFI), protein efficiency ratio (PER), body length (BL), and survival rate were determined for each tank using the following formulas:

WG (%) = [(individual final wet weight − individual initial wet weight)/initial wet weight] × 100.

SGR (%) = 100 × (Ln W2 − Ln W1)/(T2 − T1).

FE = (final weight − initial weight)/feed intake

DFI (mg·fish^−1^·day^−1^) = [Σᵢ₌1ⁿ (total feed consumption (g) × 1000/number of fish)]/number of days

PER = (final weight − initial weight)/protein intake, where protein intake = feed intake × crude protein

Survival rate (%) = 100 × (final number of fish/initial number of fish).

where Ln denotes the natural logarithm; W1 and W2 are the initial and final weights (mg), respectively; T2 − T1 is the number of experimental days.

At the end of the feeding experiment, 30 adult fish (15 females and 15 males) were randomly selected for each treatment. These individuals were distributed into three replicates per treatment, with five females and five males allocated to each aquarium. All aquaria maintained the culture conditions described for the growth trial, with the only difference being the presence of a central perforated divider that prevented direct contact between males and females until spawning. The fish remained in these systems for a period of three weeks, with spawning induced once weekly. Spawning was carried out as described by Carneiro et al. [20]

At the conclusion of the reproduction period, all females were euthanized according to the procedure described above and then dissected for gonad collection. The gonadosomatic index (GSI, %) was calculated as follows:

Gonadosomatic index (%): = (gonad weight/total body weight) × 100

The parameters assessed for reproductive performance were

Fertilization rate (%): (number of fertilized eggs/mean number of eggs per female) × 100

Hatching rate (%): (number of hatched eggs/mean number of fertilized eggs) × 100.

### 2.5. cDNA Library, Sequencing, and Data Processing

For RNA-Seq analyses, fish from the control group and the SM50 group were considered, with each group consisting of three replicates. Two fish were collected per replicate, totaling six fish per group. Muscle tissue fragments from each zebrafish sample were collected, preserved in RNA-Later (Thermo Fisher Scientific, Waltham, MA, USA), and stored at −80 °C until further analysis. RNA was isolated using the Omega E.Z.N.A.^®^ Total RNA Kit II (Omega Bio-tek, Norcross, GA, USA). Transcriptome libraries were constructed using a TruSeq RNA Library Preparation V2 kit (Illumina Inc., San Diego, CA, USA). Each sample was barcoded, resulting in a single cDNA library. Libraries were sequenced on an Illumina HiSeq 2500 platform (with 2 × 150 bp paired-end reads).

The quality of the raw data generated after sequencing was checked using FastQC software (version 0.10.1). Data were filtered with Trimmomatic v0.30, including removal of primers and adapter sequences, truncation of paired-end reads with end quality < 25, and truncation of reads with average quality below 25 in a sliding window of 4 bp based on the Phred algorithm.

### 2.6. Mapping, Identification, and Annotation of Differentially Expressed Genes

Processed reads were mapped to the zebrafish reference genome (DanrRer10) using STAR software v.2.7.5b [23]. Mapped reads were counted using FeatureCounts software v.1.6.4+galaxy2 [24]. The results were then used for the analysis of differentially expressed genes (DEGs) between SM0 and SM50 using DEseq2 software v.2.11.40.6+galaxy1 [25]. Genes with an adjusted *p*-value < 0.05 as identified by DESeq were considered differentially expressed. The false discovery rate (FDR) was adjusted using the Hochberg and Benjamini (BH) method [26]. An FDR < 0.05 and |log2 (fold change)| ≥ 1 were set as the threshold for significant differential expression.

Functional enrichment analysis of gene ontology (GO) was performed by mapping all differentially expressed genes to the Gene Ontology database. This analysis allowed for the study of gene categories and the effects of SM in the diet. Enriched GO terms were evaluated using the GOseq package v. 1.50.0 [27]. Enriched GO categories were calculated using the Wallenius approximation, and *p*-values were adjusted using the BH method. Categories with adjusted *p*-values (FDR) < 0.05 were considered enriched. To summarize the GO categories, each enriched term was annotated to a GO term linked to the root term, i.e., Cellular Component (CC), Biological Process (BP), or Molecular Function (MF). The KEGG database (Kyoto Encyclopedia of Genes and Genomes) was used to analyze relevant DEG pathways [28]; *p*-values < 0.05 were considered significantly enriched.

### 2.7. Statistical Analysis

Normality (Shapiro–Wilk test) and homoscedasticity (Levene’s test) were verified, and data were then subjected to analysis of variance (ANOVA). Significant differences among treatments were detected by Tukey’s test. A linear regression analysis was performed on growth performance data to better evaluate the relationship between fish growth and FM replacement with SM. All data were analyzed at a 95% significance level (*p* < 0.05). All statistical analyses were performed using Minitab statistical software v.18 (State College, PA, USA) and R (version 4.4.1). No animals, experimental units, or data points were excluded from the statistical analysis; all collected data were included in the analyses.

## 3. Results

### 3.1. Growth Performance

The fish readily accepted the experimental diets, and feed intake was not affected by diet composition (Table 2). Survival was not affected by the different experimental diets, remaining above 90% in all groups. At the end of the experimental period, fish performance, as evaluated by final body weight, weight gain, specific growth rate, and total body length, increased linearly with increasing levels of SM replacing FM in diets. The feed efficiency and protein efficiency ratios showed trends similar to the growth parameters.

### 3.2. Reproductive Performance

The reproductive parameters of zebrafish fed with different levels of SM replacing FM in the diet are presented in Table 3. The gonadosomatic index (GSI) was significantly higher in females fed the SM50 diet compared to the results for females in the SM0 group. Egg production was significantly higher in females fed the SM50 diet. However, no significant differences were observed in egg production per female between the SM0 diet and the SM10 and SM20 diets. The SM50 diet promoted the highest fertilization rate compared to those for the SM30 and SM10 treatments. The highest hatching rates were observed in fish fed the SM40 and SM50 diets, whereas the lowest hatching rate was found in fish fed the SM0 diet.

**Figure 1:**
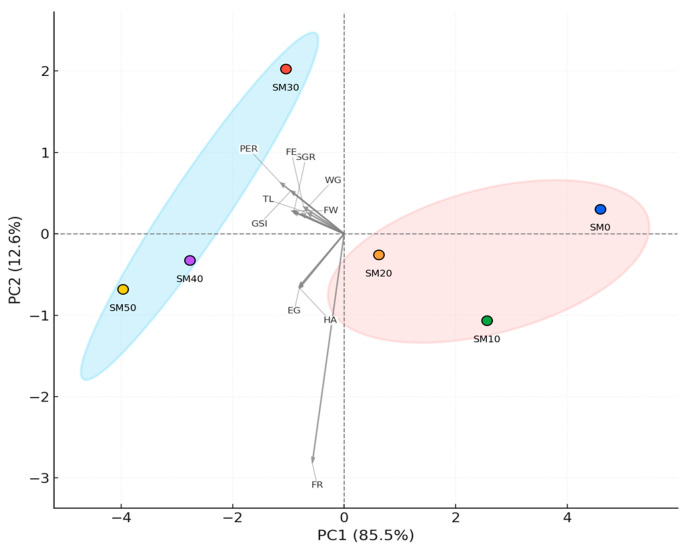
PCA biplot of growth and reproductive parameters across dietary treatments (SM0–SM50). Points denote treatments; colored ellipses show k-means clusters. Arrows indicate variable loadings: FW (final weight), WG (weight gain), SGR (specific growth rate), FE (feed efficiency), PER (protein efficiency ratio), TL (total length), GSI (gonadosomatic index), EG (egg production), FR (fertilization rate), and HA (hatching rate). PC1 and PC2 explain 85.5% and 12.6% of total variance, respectively.

Principal component analysis (PCA) accounted for 98.1% of the total variability in data across the first two components (PC1: 85.5%; PC2: 12.6%). The PCA biplot (Figure 1) revealed two main treatment clusters, delineated by the statistical (k-means) cluster ellipses. Treatments with the highest Spirulina inclusion levels (SM40 and SM50) appear in the upper-left quadrant, associated with growth performance variables (FW, WG, SGR, TL), the gonadosomatic index (GSI), egg production (EG) and hatching rate (HA). In contrast, SM0, SM10, and SM20 are grouped in the lower-right quadrant, distant from the primary variables of interest. Treatment SM30 occupied an intermediate position, suggesting transitional performance between the two groups. FW, WG, SGR, TL, GSI, EG, and HA were the variables offering the greatest contribution to the separation among experimental groups, as indicated by the magnitude and direction of the loadings on the biplot.

### 3.3. RNA-Seq Analysis

A total of 21,090,086 raw reads were obtained for the SM0 group, while 39,292,076 reads were obtained for the SM50 group. After filtering, all datasets showed an average read quality above 30 for the Phred score, indicating high confidence in the sequences obtained [29]. Subsequently, the reads were uniquely mapped to the zebrafish reference genome (GRCz10/danRer10, September 2014).

#### 3.3.1. DEGs Profiles

**Figure 2:**
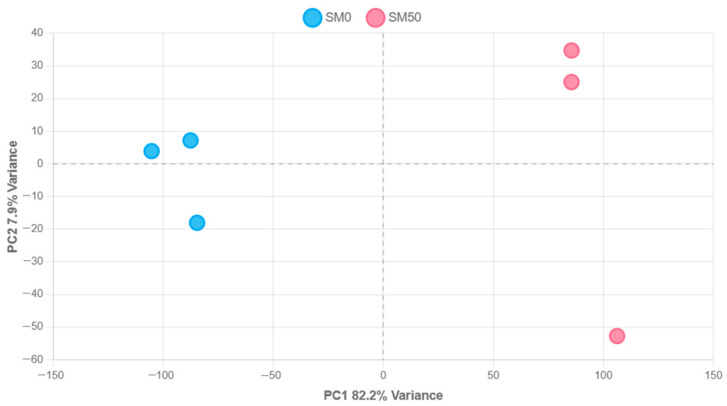
Principal component analysis (PCA) of SM0 and SM50 samples. Each circle represents an individual biological replicate (SM0_R in light-blue, *n* = 3; SM5_R in light-pink, *n* = 3). The x- and y-axes correspond to PC1 (82.2% of total variance) and PC2 (7.9% of total variance), respectively, showing clear separation between the two conditions.

After counting the mapped reads, the data were analyzed using the DESeq2 package v.2.11.40.6+galaxy1 to perform differential gene expression (DEG) analysis. Principal component analysis (PCA) of DESeq2-normalized transcriptomic data revealed a clear clustering of biological replicates within the SM0 and SM50 groups, thus demonstrating distinct transcriptional profiles between treatments (Figure 2).

**Figure 3:**
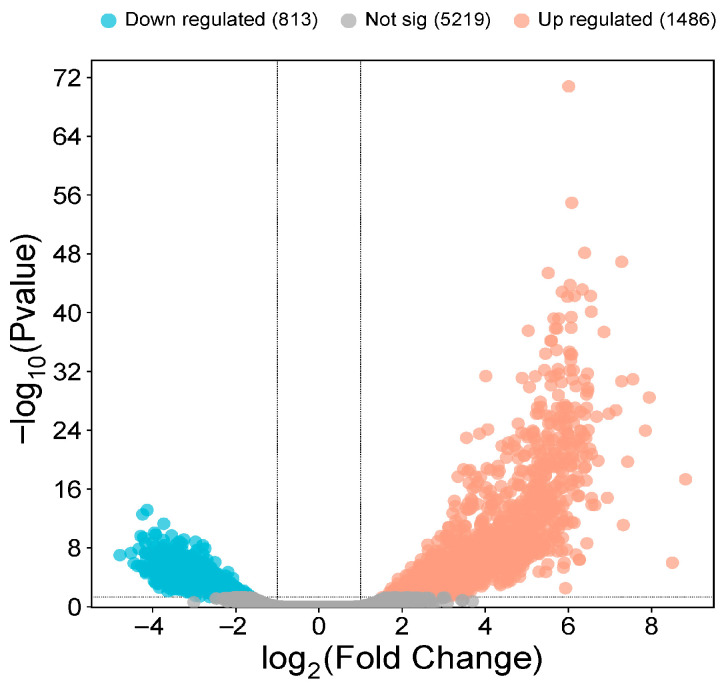
Volcano plot of SM50 vs. SM0. Down regulated genes are shown as light blue (cyan) dots (*n* = 813), up regulated genes as light orange (peach) dots (*n* = 1486), and non-significant DEGs as gray dots (*n* = 5219). The vertical dashed lines mark log_2_ (fold-change) cutoffs, and the horizontal dashed line the significance threshold (*p* = 0.05).

When comparing the SM0 and SM50 groups, 2299 differentially expressed genes were identified, of which 1486 were upregulated, and 813 were downregulated (log_2_ FC > 1) (FDR < 0.05) (Figure 3).

#### 3.3.2. Functional Annotation and Pathway Analysis of DEGs

The GO enrichment results showed that the DEGs were classified into three main functional categories: biological process (BP), molecular function (MF), and cellular component (CC).

**Figure 4:**
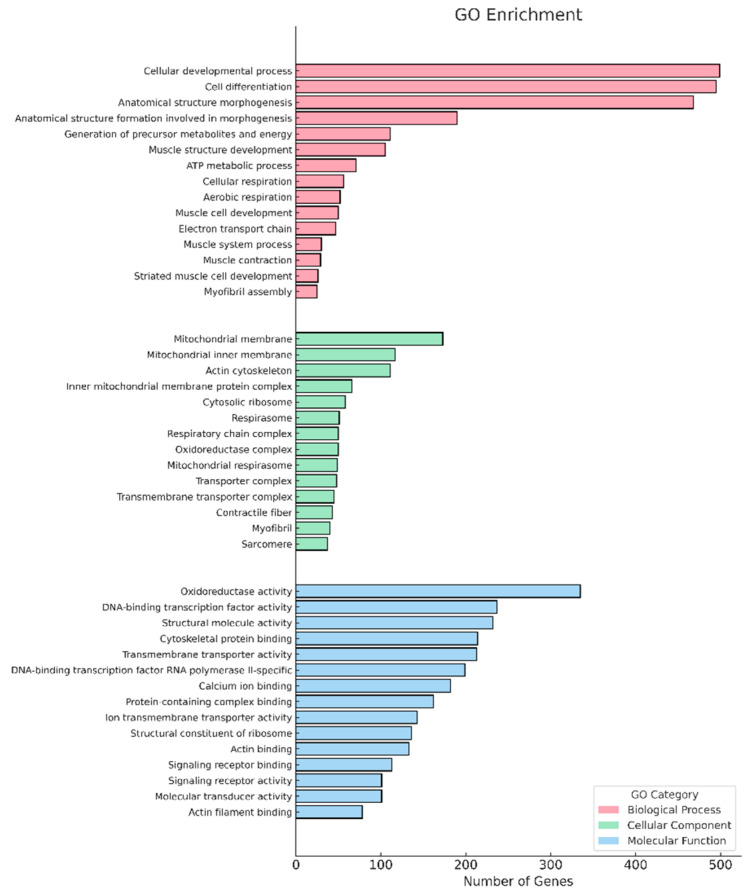
Functional analysis of DEGs based on RNA-Seq data. The results are summarized in the ten most enriched GO terms within three main categories: cellular component, biological process, and molecular function.

The top ten GO terms in each category and the respective number of genes involved in each process are shown in Figure 4. Functional enrichment analysis of the DEGs revealed a predominance of terms related to muscle development, morphogenesis and cell differentiation (GO:BP); sarcomere structure and mitochondria (GO:CC); and transcription factors, oxidoreductase activity, and calcium binding (GO:MF). The main terms included the cellular developmental process (499 genes), the mitochondrial membrane (173 genes), and oxidoreductase activity (335 genes).

**Figure 5:**
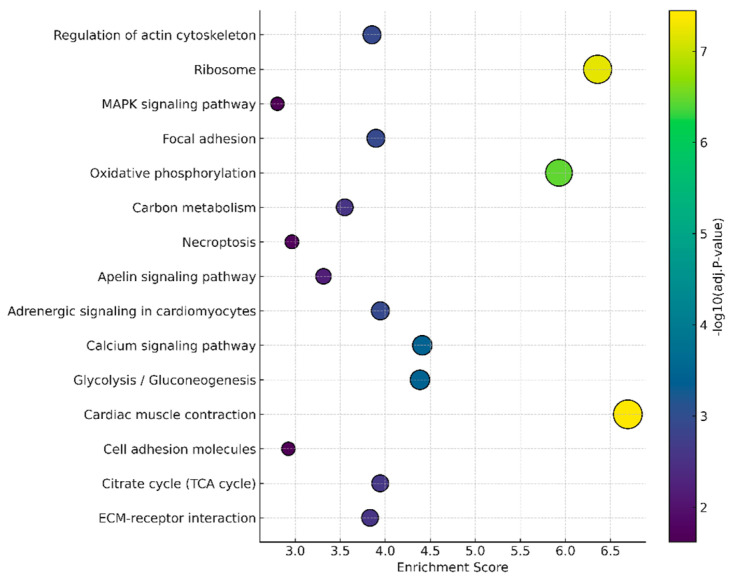
Bubble plot representing the main enriched pathways according to the KEGG analysis. The Y-axis indicates the enriched pathways, while the X-axis represents the enrichment score.

Functional enrichment analysis based on KEGG pathways revealed a set of significantly modulated pathways among the upregulated genes in the SM50 group (Figure 5). The main enriched pathways included those directly related to muscle and energy activity, such as cardiac muscle contraction, oxidative phosphorylation, glycolysis/gluconeogenesis, and the calcium signaling pathway.

**Figure 6:**
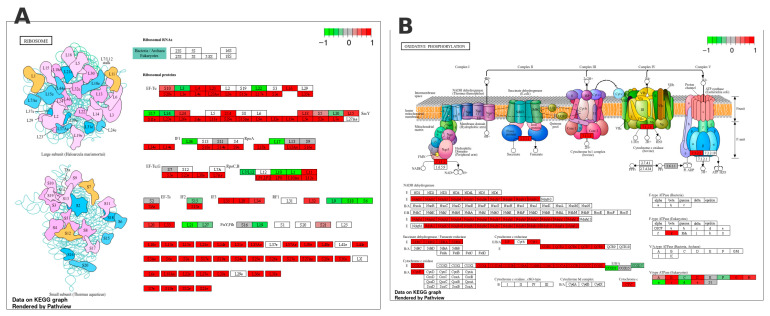
KEGG pathway maps show differential gene expression in SM50 vs. SM0. (**A**) Ribosome: maps of the large (top) and small (bottom) ribosomal subunits. (**B**) Oxidative phosphorylation: complexes I–V of the mitochondrial electron-transport chain. In both panels, genes are colored according to their log_2_(fold-change): shades of red denote upregulation, shades of green denote downregulation, and gray denotes non-significant changes. The color bar in the upper right shows the log_2_ (fold-change) scale.

Among the most significantly enriched KEGG pathways, the ribosome pathway stood out (Figure 6A), with a predominance of upregulated ribosomal protein-coding genes, covering both large and small ribosomal subunits. In addition, the oxidative phosphorylation pathway also showed significant enrichment (Figure 6B), with predominant upregulation of genes related to subunits of complexes I, III, IV, and V of the mitochondrial electron transport chain. This pattern indicates an intensification of ATP production via oxidative metabolism, meeting the high energy demand associated with protein biosynthesis and muscle development.

## 4. Discussion

### 4.1. Growth Performance

The results of the present study show that complete replacement of FM with Spirulina significantly increased the final weight, specific growth rate, and protein efficiency ratio in zebrafish, with no mortality or clinical signs throughout the trial. These findings indicate its safety and acceptability under the tested conditions and are consistent with reports of growth-promoting effects in several fish species [30,31,32].

Responses to Spirulina, however, are species-dependent. In catla (*Catla catla*), complete replacement of FM with Spirulina, equivalent to approximately 30% dietary inclusion, had a neutral effect on growth [33]. Similarly, in common carp (*Cyprinus carpio*), complete replacement of FM with Spirulina did not change growth performance [34]. In contrast, in silver seabream (*Rhabdosargus sarba*), growth decreased with 75% FM replacement and showed a significantly greater reduction at 100%; these outcomes were accompanied by reduced feed intake at higher Spirulina levels, suggesting limitations in palatability and/or digestibility [35]. Moreover, in that seabream study, Spirulina was the sole protein source in the 100% FM-replacement diet, whereas in the present study, despite complete FM replacement, Spirulina accounted for 5% of the total diet, which may mitigate antinutritional or palatability issues associated with high inclusion levels.

The increase in growth performance observed in this study may be attributed to several mechanisms. First, the amino acid profile of Spirulina, containing all essential amino acids [36], can support protein synthesis and muscle anabolism, consistent with the higher PER observed. Second, its lipid composition, together with bioactive pigments such as phycocyanin and carotenoids, display antioxidant activity that may protect hepatocytes and optimize hepatic metabolism, thereby improving protein utilization [37]. In addition, phycocyanin has been associated with the increased activity of digestive enzymes, which can facilitate digestion and nutrient absorption [38]. Spirulina has also been reported to modulate the gut microbiota, promoting bacteria involved in peptide hydrolysis and bile acid metabolism, potentially increasing nutrient absorption [39,40]. These mechanisms explain the observed pattern where FW, WG, SGR, FE, and PER improved from SM30 onwards, with the highest values generally recorded for SM40 and SM50.

### 4.2. Reproductive Performance

The success of fish reproduction is influenced by various factors, such as broodstock nutrition, feeding rates, stocking density, age, and size [41,42]. In the present study, the reproductive performance of zebrafish was significantly affected by the replacement of FM with Spirulina in the diets. The gonadosomatic index of females fed the SM50 diet (10.1%) was significantly higher than that observed in females from the SM0 group (6.31%). In addition, an approximately 2.5-fold increase in egg production was observed in females fed the diet containing 50 g kg^−1^ Spirulina compared to females from the SM0 group. These results are consistent with those reported by James et al. [43], who found that female red swordtail (*Xiphophorus helleri*) showed an increase in GSI in response to increased dietary Spirulina levels. The same authors observed that fish fed a diet containing 8% Spirulina displayed a gonad weight up to four times greater than that of fish fed diets with 0% and 3% Spirulina.

The significant improvements in GSI, egg production, and hatching rate observed in the groups fed Spirulina (SM40 and SM50) indicate a substantial enhancement in reproductive performance. These observations are consistent with the results of previous studies that have identified underlying physiological mechanisms. For instance, Calabrò et al. [44] reported that Spirulina supplementation not only accelerated sexual maturation but also significantly increased the expression of vitellogenin (vtg), the yolk precursor protein. Complementing this, Coli et al. [30] observed a significant increase in the mean diameter of stage IV oocytes with a 4–8% Spirulina diet, without changes in the distribution of maturation stages, reflecting enhanced vitellogenin deposition.

While these studies identified key physiological steps, our findings substantiate a causal link between these mechanisms and measurable reproductive outcomes. Specifically, the enhanced vitellogenesis documented herein correlates with elevated GSI indices, improved egg production, and consequently, the highest hatching rates. This positive effect of Spirulina on hatching is not exclusive to zebrafish, as similar results have been documented in other species such as the yellow-tail cichlid [45].

Integrating our findings with those of Calabrò et al. [44] and Coli et al. [30], our results support the efficacy of Spirulina as a functional ingredient in zebrafish broodstock diets. The primary mechanism appears to involve enhanced vitellogenesis, likely mediated by the carotenoid and antioxidant constituents of the microalga.

Multivariate principal component analysis highlighted these improvements, demonstrating that treatments with the highest Spirulina inclusion levels (SM40, SM50) are significantly correlated with enhanced growth metrics (FW, WG, SGR, TL) and reproductive parameters (GSI, egg production, hatching rate). Such effects can be attributed to Spirulina’s composition, including phycocyanin, β-carotene, essential fatty acids, vitamins, and minerals, which are likely to support anabolic processes, gonadal development, and gamete viability through antioxidant and immunomodulatory mechanisms [31,46]. These findings are consistent with those of previous studies in various fish species [30,31,45,46], supporting the inclusion of Spirulina as a functional dietary ingredient to improve reproductive performance and overall growth.

### 4.3. Differential Gene Expression

Fish health and proper growth are directly linked to the quality of nutrients consumed by these animals. Understanding the impacts of nutrition on fish growth and immunity is increasingly important due to the growing number of fish maintained on formulated diets [47,48].

Transcriptome sequencing is a valuable tool for characterizing gene expression underlying the mechanisms involved in growth, reproduction, immunity, and stress in fish [47,48,49]. In the present study, transcriptome sequencing identified 2299 DEGs, which were grouped according to the corresponding GO terms.

When analyzing the differential gene expression profile of fish fed Spirulina, a positive regulation of important genes related to muscle development and functionality in zebrafish was identified. In the GO BP category, factors such as muscle structure development, cell differentiation, and muscle cell development were significantly enriched, indicating the activation of pathways associated with the formation and maintenance of muscle tissue.

GO analysis revealed the activation of muscle development processes, while KEGG analysis showed upregulation of central metabolic pathways. These complementary findings indicate a coordinated anabolic response in zebrafish supplemented with Spirulina. In particular, the enrichment of the ribosome pathway indicates an increase in translational capacity, a fundamental condition for supporting the synthesis of structural proteins, growth, and survival [50,51]. Additionally, the oxidative phosphorylation pathway was significantly activated, indicating an intensification of ATP production, which is essential to meet the energy demand during growth and muscle differentiation [52]. The glycolysis/gluconeogenesis pathway was also enriched, indicating an increase in glycolytic flux to provide energy and metabolic intermediates necessary for the biosynthesis of cellular components.

In addition, Spirulina supplementation in zebrafish upregulated pathways fundamental to muscle growth and cellular energetics (ribosome/translation, oxidative phosphorylation, sarcomere organization, calcium signaling, and glycolysis/gluconeogenesis), supporting its use as a functional ingredient with potential benefits for production efficiency and fish health. Because these axes are conserved across teleosts [18,53], the mechanisms identified are likely applicable to commercial species such as Nile tilapia, common carp, and salmonids. Selected transcripts or proteins from these pathways could serve as candidate biomarkers to guide diet formulation and monitor nutritional status in production settings. Cross-species validation should define dose–response and long-term effects, including functional performance and stress resilience, under relevant environmental conditions.

## 5. Conclusions

In conclusion, Spirulina can completely replace fish meal (50 g kg^−1^) in zebrafish diets without adverse effects on growth. This replacement led to superior performance, characterized by significant increases in growth, protein efficiency, egg production, fertilization rate, and hatching rate. Transcriptomic analysis revealed the activation of key pathways associated with protein synthesis, energy metabolism, and muscle development. This study reinforces the potential of Spirulina as a functional ingredient with high biological value in aquaculture, capable of reducing reliance on fish meal.

## Figures and Tables

**Table 1:** Ingredients (g kg^−1^) and proximate composition (wet weight) of diets for juvenile zebrafish (*Danio rerio*) with increasing levels of Spirulina replacing fish meal.

Ingredients	Diet Designation (g kg^−1^ Fish Meal Replacement)					
	0	10	20	30	40	50
Fish meal ^1^	50	40	30	20	10	0
Soybean meal ^2^	594.3	593.4	594.5	595.6	596.7	597.8
Spirulina ^3^	0	10	20	30	40	50
Corn meal ^2^	312.4	323.4	318.2	313	307.7	302.5
Dicalcium phosphate ^4^	26	22.2	23.5	24.8	26.12	27.4
Premix ^5^	5	5	5	5	5	5
Salt ^4^	5	5	5	5	5	5
Limestone ^4^	3	0.27	0.64	1	1.37	1.74
Soybean oil ^4^	4.1	0.58	3	5.4	7.88	10.3
BHT ^6^	0.2	0.2	0.2	0.2	0.2	0.2
Analyzed composition ^7^ g kg^−1^						
Dry matter	887	886	879	889	879	889
Crude protein	323	322	321	325	321	326
Ether extract	34	53	41	42	42	48
Ash	67	66	57	65	57	72
Gross energy (MJ kg^−1^)	17.01	17.42	17.19	17.26	17.21	17.28

^1^ Total Alimentos (Archer Daniels Midland Company)—Três Corações, MG, Brazil. ^2^ GEM animal nutrition—Acreúna, GO, Brazil. ^3^ Proximate composition (dry-matter basis, g kg^−1^): dry-matter 877; crude protein 723; ether extract 29.5; ash 47. ^4^ All ingredients were obtained from a local commercial source. ^5^ Guaranteed vitamin and mineral supplement levels per kilogram of product: vit. A = 1,200,000 IU; vit. D3 = 200,000 IU; vit. E = 12,000 mg; vit. K3 = 2400 mg; vit. B1 = 4800 mg; vit. B2 = 4800 mg; vit. B6 = 4000 mg; vit. B12 = 4800 mg; folic acid = 1200 mg; calcium pantothenate = 12,000 mg; vit. C = 48,000 mg; biotin = 48 mg; choline = 65,000 mg; niacin = 24,000 mg; Fe =10,000 mg; Cu = 6000 mg; Mn = 4000 mg; Zn = 6000 mg; I = 20 mg; Co = 2 mg; Se = 20 mg. ^6^ BHT = butyl hydroxy toluene. ^7^ Diets were analyzed by the ESALQLAB (Universidade de São Paulo).

**Table 2:** Growth performance and survival of juvenile zebrafish (*Danio rerio*) after nine weeks of feeding with different levels of *Arthrospira platensis* as a replacement for fish meal.

Parameters	Diets						Linear Regression	
	SM0	SM10	SM20	SM30	SM40	SM50	*p*-Value	R^2^
IW	160.46 ± 0.17	159.98 ± 0.51	159.80 ± 1.14	160.33 ± 0.21	160.56 ± 0.13	159.74 ± 0.66		
FW	391.57 ± 2.10 ^d^	412.31 ± 15.48 ^cd^	444.38 ± 13.77 ^bc^	470.60 ± 17.23 ^ab^	477.12 ± 11.99 ^ab^	499.84 ± 10.99 ^a^	<0.01	0.78
WG	149.82 ± 4.43 ^c^	157.79 ± 10.26 ^bc^	187.89 ± 11.89 ^abc^	182.86 ± 11.07 ^abc^	197.16 ± 7.49 ^ab^	212.85 ± 5.62 ^a^	<0.01	0.77
SGR	1.49 ± 0.10 ^d^	1.57 ± 0.07 ^cd^	1.70 ± 0.05 ^bc^	1.79 ± 0.01 ^ab^	1.81 ± 0.04 ^ab^	1.90 ± 0.03 ^a^	<0.01	0.78
FE	0.42 ± 0.02 ^b^	0.46 ± 0.03 ^ab^	0.51 ± 0.03 ^ab^	0.55 ± 0.02 ^a^	0.56 ± 0.03 ^a^	0.57 ± 0.02 ^a^	<0.01	0.52
DFI	9.17 ± 0.40	9.25 ± 0.55	9.34 ± 0.58	9.36 ± 0.27	9.44 ± 0.48	10.04 ± 0.50	0.82	0.10
PER	1.29 ± 0.05 ^b^	1.39 ± 0.10 ^ab^	1.59 ± 0.10 ^ab^	1.70 ± 0.06 ^a^	1.75 ± 0.11 ^a^	1.74 ± 0.06 ^a^	<0.01	0.53
Survival	93.33 ± 5.44	91.67 ± 10	95.00 ± 6.38	95.00 ± 6.38	93.33 ± 9.43	95.00 ± 6.38	0.86	0.10
TL	31.81 ± 0.22 ^c^	32.16 ± 0.26 ^c^	33.19 ± 0.61 ^bc^	35.28 ± 0.42 ^ab^	36.36 ± 0.51 ^a^	36.67 ± 0.51 ^a^	<0.01	0.86

Data are expressed as mean ± standard error of the mean. Means in the same row with different superscript letters are significantly different (*p* < 0.05) according to analysis of variance and Tukey’s test (*p* < 0.05). FW = final weight (mg); WG= weight gain (%); SGR = specific growth rate (%); FE = feed efficiency; DFI = feed intake (mg·fish^−1^·day^−1^); PER = protein efficiency ratio %; survival = final mean survival %; TL = total length, mm.

**Table 3:** Reproductive parameters of zebrafish (*Danio rerio*) fed different levels of *Arthrospira platensis* as a replacement for fish meal for nine weeks.

Diets	Gonadosomatic Index (%)	Egg Production	Fertilization Rate (%)	Hatching Rate (%)
SM0	6.31 ± 0.73 ^b^	101.11 ± 10.07 ^c^	77.43 ± 0.35 ^bc^	58.60 ± 4.43 ^b^
SM10	7.01 ± 0.80 ^ab^	125.67 ± 4.17 ^c^	85.37 ± 1.76 ^ab^	65.97 ± 3.02 ^ab^
SM20	7.50 ± 0.86 ^ab^	124.56 ± 3.97 ^c^	84.46 ± 0.20 ^abc^	68.65 ± 3.90 ^ab^
SM30	9.36 ± 0.72 ^ab^	144.22 ± 8.19 ^bc^	75.97 ± 2.11 ^c^	68.25 ± 1.63 ^ab^
SM40	9.58 ± 0.31 ^ab^	220.01 ± 37.29 ^ab^	86.40 ± 3.29 ^ab^	75.53 ± 2.56 ^a^
SM50	10.01 ± 0.77 ^a^	246.56 ± 17.03 ^a^	88.41 ± 1.87 ^a^	78.97 ± 0.89 ^a^
Linear Regression				
*p*-value	<0.01	<0.01	<0.01	<0.01
R^2^	0.61	0.72	-	0.66

Data are expressed as mean ± standard error of the mean. Means in the same column with different superscript letters are significantly different (*p* < 0.05) based on analysis of variance and Tukey’s test (*p* < 0.05).

## Data Availability

The data presented in this study are available on request from the corresponding author.
